# Evaluation of Toll-like Receptor 4 (TLR4) Involvement in Human Atrial Fibrillation: A Computational Study

**DOI:** 10.3390/genes15050634

**Published:** 2024-05-16

**Authors:** Paolo Fagone, Katia Mangano, Maria Sofia Basile, José Francisco Munoz-Valle, Vincenzo Perciavalle, Ferdinando Nicoletti, Klaus Bendtzen

**Affiliations:** 1 Department of Biomedical and Biotechnological Sciences, University of Catania, Via S. Sofia 89, 95123 Catania, Italy; 2 Faculty of Medicine, Kore University, 94100 Enna, Italy; 3 Institute for Research in Biomedical Sciences, University Center for Health Sciences, University of Guadalajara, Guadalajara 44100, Jalisco, Mexico; 4 Institute for Inflammation Research, Rigshospitalet University Hospital, 2100 Copenhagen, Denmark

**Keywords:** atrial fibrillation, Toll-like receptors, TLR4, computational study

## Abstract

In the present study, we have explored the involvement of Toll-like Receptor 4 (TLR4) in atrial fibrillation (AF), by using a meta-analysis of publicly available human transcriptomic data. The meta-analysis revealed 565 upregulated and 267 downregulated differentially expressed genes associated with AF. Pathway enrichment analysis highlighted a significant overrepresentation in immune-related pathways for the upregulated genes. A significant overlap between AF differentially expressed genes and TLR4-modulated genes was also identified, suggesting the potential role of TLR4 in AF-related transcriptional changes. Additionally, the analysis of other Toll-like receptors (TLRs) revealed a significant association with TLR2 and TLR3 in AF-related gene expression patterns. The examination of MYD88 and TICAM1, genes associated with TLR4 signalling pathways, indicated a significant yet nonspecific enrichment of AF differentially expressed genes. In summary, this study offers novel insights into the molecular aspects of AF, suggesting a pathophysiological role of TLR4 and other TLRs. By targeting these specific receptors, new treatments might be designed to better manage AF, offering hope for improved outcomes in affected patients.

## 1. Introduction

TLRs were initially identified as Toll proteins that mediate innate immunity in the fruit fly *Drosophila melanogaster*. TLRs constitute a family of pattern recognition receptors (PRRs) capable of recognizing pathogen-associated molecular patterns (PAMPs) as well as molecules released by damaged cells termed damage-associated patterns (DAMPs) [1,2,3,4]. In the human heart, TLRs, particularly TLR4, TLR2, and TLR3, exhibit varying expression levels. TLR4, the first characterized mammalian Toll protein, is secreted from the endoplasmic reticulum (ER) and trafficked to the Golgi compartment called cisternae, ultimately reaching the plasma membrane. Activation of TLR4 by endotoxic bacterial lipopolysaccharide (LPS) involves cofactors like CD14, MD-2, and LBP, leading to the recognition of various ligands, including PAMPs and DAMPs. Recent studies propose macrophage scavenger receptor class A as a co-receptor for TLR4, influencing inflammatory responses. 

Upon activation by lipopolysaccharides (LPS), TLR4 triggers two main signalling pathways: the MyD88-dependent and the MyD88-independent pathways. In the MyD88-dependent pathway, TLR4 recognition of LPS facilitates the recruitment of the adaptor protein MyD88, which then attracts IL-1 receptor-associated kinases (IRAKs), including IRAK4 and IRAK1. These kinases subsequently activate TRAF6 (TNF receptor-associated factor 6), which in turn interacts with TAK1 and its associated proteins TAB1 and TAB2, leading to the activation of the IKK complex. This complex phosphorylates the inhibitor IκBα, prompting its degradation and thereby allowing NF-κB to translocate into the nucleus and initiate transcription of pro-inflammatory cytokines, such as IL-6, IL-1β, and TNF-α. In parallel, the MyD88-independent pathway operates concurrently. TRIF recruitment to TLR4 leads to the activation of TBK1 and IKKε, which phosphorylate interferon regulatory factor 3 (IRF3). Phosphorylated IRF3 dimerizes and moves to the nucleus to promote the production of type I interferons. Additionally, this pathway can also result in the delayed activation of NF-κB via TRIF, contributing further to the inflammatory responses by enhancing cytokine production at a later stage. Interestingly, TLR4 and the PI3K/Akt signalling pathway appear to counter-regulate each other, although the specific mechanisms remain unclear [1,2,3,4].

TLR4, in association with MD-2, detects lipopolysaccharides (LPSs) from Gram-negative bacteria like Klebsiella and Escherichia, which are implicated in conditions such as human atrial fibrillation (AF) [5,6,7,8]. Additionally, TLR4 is critically involved in inflammatory responses in various cardiac conditions [9,10,11,12,13,14]. In viral myocarditis caused by Coxsackievirus B3, TLR4-deficient mice show reduced inflammation and myocarditis due to decreased viral replication. TLR4 also affects myocardial infarction (MI) by mediating inflammatory responses through damage-associated molecular patterns from necrotic cardiac cells. Inhibition of TLR4 signalling, such as with metformin, can reduce inflammation and improve cardiac outcomes. Similarly, TLR4 inhibition has been shown to alleviate the effects of myocardial ischaemia/reperfusion injury, highlighting its multifaceted role in cardiac pathology [9,10,11,12,13,14].

Based on the aforementioned considerations, we have conducted a computational study to assess the potential involvement of the TLR4 pathway in AF. This condition stands as the most prevalent and persistent global heart rhythm disorder. Its most serious consequence is that it elevates the risk of thromboembolism due to blood clot formation, leading to a nearly 5-fold increased risk of debilitating stroke and other cardiovascular complications, including stroke [15,16,17,18,19].

The availability of gene expression profile data has prompted the use of bioinformatics methods as powerful tools to in-depth explore pathophysiological processes. In this study, we utilized publicly available gene microarray data to conduct a meta-analysis, aiming at characterizing whether TLR4 is implicated in the transcriptional changes occurring in the cardiac tissue of AF patients.

## 2. Materials and Methods

### 2.1. Dataset Selection and Analysis

The gene expression profile data were obtained from the Gene Expression Omnibus (GEO) database of the National Center for Biotechnology Information (NCBI) at https://www.ncbi.nlm.nih.gov (accessed on 2 January 2024). The keyword search included “atrial fibrillation” AND “homo sapiens”. Twenty-nine series were identified, and among them, expression data from the left atrial appendage (LAA) were selected. Based on these criteria, five datasets (GSE79768 [20], GSE31821, GSE128188 [21], GSE14975 [22], and GSE41177 [23]) were chosen. GSE79768 included 7 samples from patients with AF and 6 samples from patients without AF. GSE31821 included 2 cases from AF patients and 2 from SR patients. In GSE128188, there were 5 patients with AF and 5 without AF. GSE14975 comprised 5 atrial tissue samples from patients with AF and 5 from patients without AF. GSE41177 included 16 samples from AF patients and 3 samples from SR patients. 

The Linear Model for Microarray Analysis (LIMMA) algorithm, implemented in the GEOexplorer web-based tool (https://geoexplorer.rosalind.kcl.ac.uk/ accessed on 2 January 2024) [24], was used for the identification of the differentially expressed genes (DEGs). An FDR of <0.05 was used as a threshold to select the DEGs. In instances where multiple microarray probes mapped the same NCBI GeneID, we selected probes with the lowest *p* value. For the meta-analysis of the datasets, we employed the Fisher’s Inverse χ2 test. The AF gene signature was finally defined by selecting genes with an FDR of <0.05 and a Median |log_2_ (Fold Change)| of >0.5.

### 2.2. Functional Analysis of Differentially Expressed Genes (DEGs) and Assessment of TLR4 Involvement in AF

Following the identification of DEGs, we conducted a functional annotations analysis using the EnrichR Web Server (https://maayanlab.cloud/Enrichr/; accessed on 5 February 2024) [25]. The gene-set libraries employed for this analysis included Reactome_2022, KEGG_2021_Human, and LINCS_L1000_CRISPR_KO_Consensus_Sigs. The gene-set libraries used in EnrichR represent curated collections of gene sets that are associated with specific biological pathways, functions, or experimental conditions. Reactome is a freely available, open-source pathway database that provides curated and peer-reviewed pathways for human biology [26]. The Reactome_2022 library includes gene sets representing various biological processes, signalling pathways, and molecular interactions as defined in the Reactome database for the year 2022. KEGG (Kyoto Encyclopedia of Genes and Genomes) is a database that integrates genomic, chemical, and systemic functional information [27]. The KEGG_2021_Human library consists of gene sets related to pathways, diseases, and drugs, with a focus on human biological systems, as of the year 2021. Library of Integrated Network-Based Cellular Signatures (LINCS) [28] is a consortium that provides gene expression signatures resulting from various cellular perturbations, including CRISPR knockout experiments. The LINCS_L1000_CRISPR_KO_Consensus_Sigs gene-set library contains consensus signatures derived from CRISPR knockout experiments on different cells lines for each gene within the LINCS framework. These combined data signatures represent a standardized response across multiple experiments or conditions, allowing the identification of robust patterns in gene expression changes due to specific gene knockouts [28].

### 2.3. Statistical Analysis

For the evaluation of the involvement of TLRs in atrial fibrillation (AF), we considered the LINCS L1000 CRISPR KO consensus signature for each TLR, as well as for MYD88 and TICAM1. EnrichR employs Fisher’s exact test to calculate the *p*-value, and it utilizes the Benjamini–Hochberg method for correcting the testing of multiple hypotheses. The z-score, which assesses the deviation from the expected rank, is computed through a modification of the Fisher exact test. The Combined Score is then determined by combining the *p*-value and the z-score (Combined Score = ln(*p*-value) × z-score).

The Cytoscape software (v. 3.10.2) [29] was used for the visualization of the network, using the STRING database to define the protein–protein interactions. Volcano plot, Enrichment Bubble plots, UpSet plots, and KEGG pathway map were generated using the online platform SRplot (https://www.bioinformatics.com.cn/srplot, accessed on 10 February 2024). Venn diagrams were generated using the web-based application Venny (https://bioinfogp.cnb.csic.es/tools/venny/; accessed on 10 February 2024).

## 3. Results

### 3.1. Meta-Analysis Results and Pathway Enrichment Analysis

To assess the involvement of Toll-like Receptor 4 (TLR4) in atrial fibrillation (AF), we conducted a comprehensive analysis using data retrieved from the Gene Expression Omnibus (GEO) database. The primary objective was to identify whole-genome transcriptomic datasets that could provide insights into the gene expression profile associated with AF. Specifically, we focused on selecting datasets that represented mRNA expression levels from the left atrial appendage of persistent AF patients compared to control patients with sinus rhythm.

**Figure 1:**
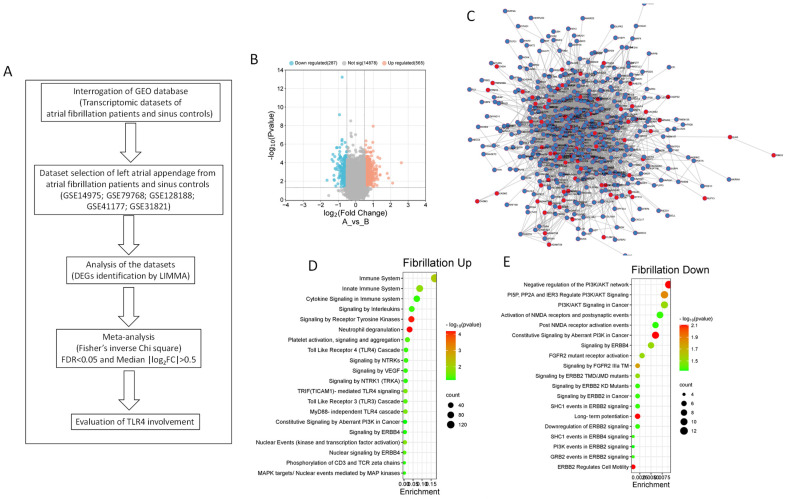
Experimental study layout (**A**). Volcano plot showing the DEGs characterizing AF (**B**). Gene Network for the AF DEGs. Upregulated DEGs are shown in blue, while downregulated DEGs are shown in red (**C**). Bubble plot showing the Reactome pathway enrichment analysis for the up-regulated DEGs in AF (**D**). Bubble plot showing the Reactome pathway enrichment analysis for the downregulated DEGs in AF (**E**).

Aiming to capture a robust representation of gene expression patterns in AF, five datasets meeting the specified criteria were chosen for the analysis. By employing the Fisher inverse chi-square method, we performed a meta-analysis on these datasets to reveal the global changes associated with AF. The overall study design is presented in Figure 1A.

Upon conducting the meta-analysis, we identified 565 differentially upregulated DEGs and 267 differentially downregulated DEGs, as shown in Figure 1B,C and File S1. 

Pathway enrichment analysis using the Reactome database revealed a comprehensive overview of pathways that were significantly overrepresented among both upregulated and downregulated DEGs. Among the upregulated DEGs, a significant enrichment was observed in pathways closely linked to the immune system. Specifically, pathways such as “Immune System”, “Innate Immune System”, “Cytokine Signalling in Immune System”, and “Signalling by Interleukins” exhibited a significant overrepresentation. Additionally, there was a significant enrichment for pathways related to cellular communication and response, including “Signalling by Receptor Tyrosine Kinases”, “Neutrophil Degranulation”, “Platelet Activation, Signalling, and Aggregation”, “Toll-like Receptor 4 Cascade”, “Signalling by NTRKs”, and “Signalling by VEGFs” (Figure 1D).

Conversely, the downregulated DEGs showed enrichment in pathways associated with the negative regulation of cellular processes. Noteworthy enrichment pathways included “Negative Regulation of the PI3K/Akt Network”, “PI3P, PP2A, and IER3 Regulate PI3K/AKT Signalling”, and “PI3K/AKT Signalling in Cancer” (Figure 1D).

### 3.2. In-Depth Exploration of the Involvement of TLR4 Signalling in AF

**Figure 2:**
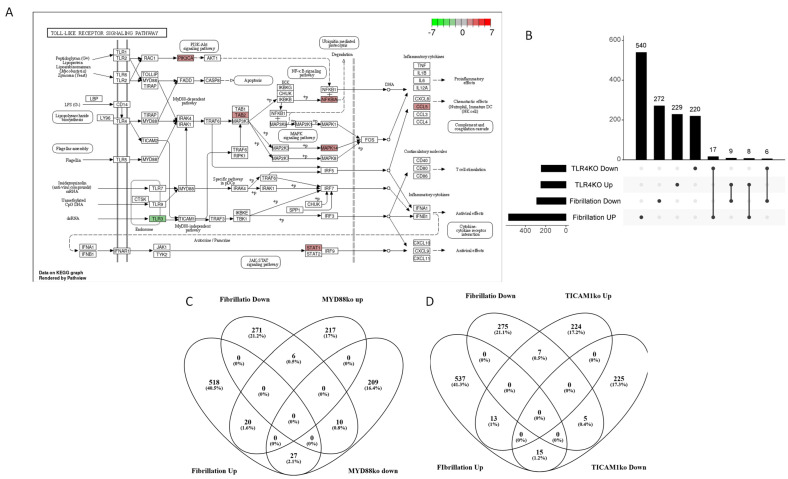
Involvement of TLR4 in atrial fibrillation. KEGG Toll-like receptor signalling pathway with genes colour-coded based on their modulation in AF (**A**). UpSet plot showing the overlap between the AF gene signature and the TLR4 consensus signature (**B**). Venn diagram showing the overlap between the AF gene signature and the consensus signatures for MYD88 (**C**). Venn diagram showing the overlap between the AF gene signature and the consensus signatures for TICA; M1 (**D**).

Subsequently, we aimed to investigate the involvement of TLR4 signalling in AF. In Figure 2A, the KEGG Toll-like receptor signalling pathway is depicted, with AF DEGs colour-coded based on their median fold change. To highlight the potential implication of TLR4 signalling in the transcriptional alterations observed in atrial tissue from AF patients, we extracted the consensus transcriptional profile derived from TLR4 knockdown cells, available in LINCS1000. As depicted in Figure 2B and detailed in Table 1, a significant number of AF DEGs exhibit a consensual modulation with transcripts that are modulated upon the genetic manipulation of TLR4. 

Given that TLR4 signals through both a MYD88-dependent and a MYD88-independent pathway (mediated by TRIF, encoded by the *TICAM1* gene), we conducted a comprehensive assessment of their roles in AF. By using the consensus gene profile from L1000, we sought to elucidate how both MYD88 and TICAM1 contribute to the transcriptional changes observed in atrial tissues of AF patients. As depicted in Figure 2C,D, and in Table 2, our findings revealed a significant yet non-specific enrichment of AF differentially expressed genes associated with both MYD88 and TICAM1. These data provide evidence that a significant number of genes regulated by MYD88 and TICAM1 have a consensual modulation in the context of AF. On the other hand, our analysis also revealed a significant number of MYD88- and TICAM1-related genes with an opposite regulation in AF (Figure 2C,D).

**Figure 3:**
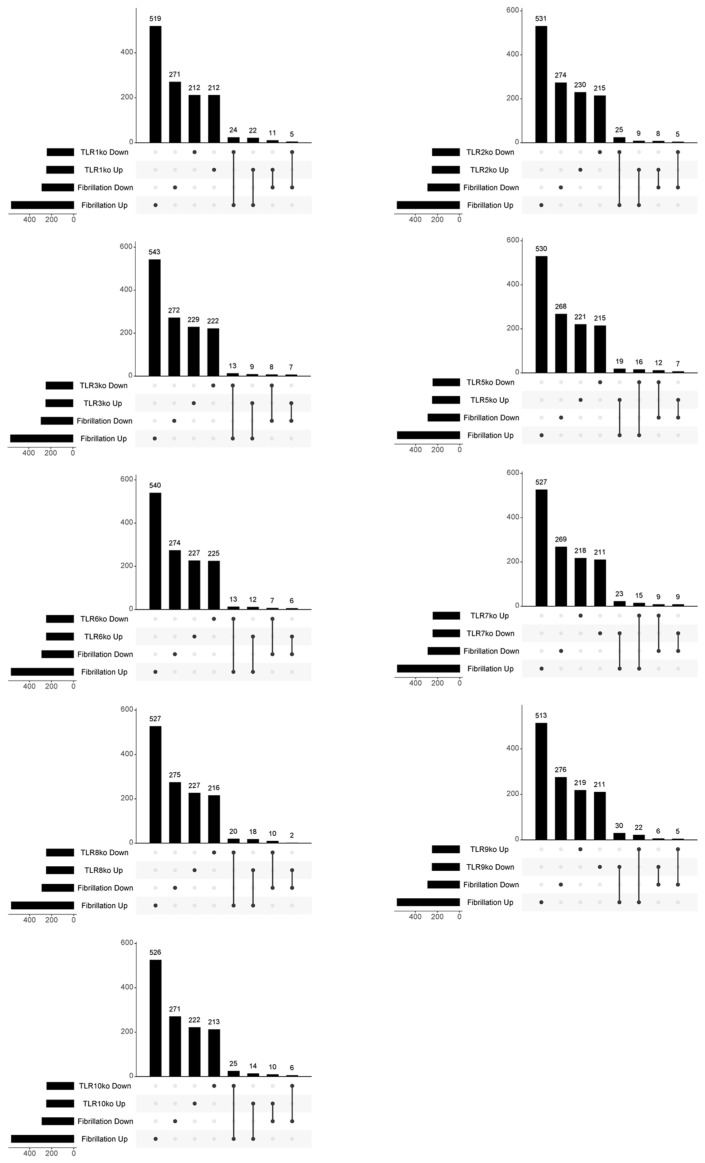
UpSet plots showing the overlap between the AF gene signature and the consensus signatures for the TLRs.

Expanded analyses of other Toll-like receptors (TLRs) are shown in Figure 3 and in Table 3. A significant number of DEGs demonstrated overlap with the consensus gene signatures of various TLRs exhibiting both concordant and discordant regulation patterns. Notably, a more distinct and specific association was identified for TLR2 and TLR3. Specifically, 25 out of 245 downregulated genes upon TLR2 knockdown were shared with AF-upregulated DEGs (*p* < 0.001), and 13 out of 247 downregulated genes upon TLR3 knockdown were shared with AF-upregulated DEGs (*p* = 0.0241).

## 4. Discussion

The altered regulation of the TLR4 signalling pathway has been associated with a wide range of conditions, including sepsis, acute lung injury, acute kidney injury, rheumatoid arthritis, inflammatory bowel disease, cardiac diseases, diabetes-induced high blood pressure, pregnancy-related disorders, and complications following COVID-19 [30].

A comprehensive understanding of the multifaceted implications of TLR4 dysregulation in these contexts is imperative for advancing targeted therapeutic interventions and elucidating potential avenues for preventive strategies across this spectrum of health challenges.

Globally, AF stands as the predominant sustained arrhythmia, and it ranks among the primary causes of stroke, heart failure, sudden death, and cardiovascular morbidity. The estimated overall count of AF patients has reached 34 million and is steadily increasing in part due to population ageing [15,16,17,18,19]. Despite its prevalence, the pathophysiologic mechanisms underlying many AF cases remain elusive, leading to a lack of effective treatment options. Catheter ablation or cardiac surgery can only restore normal heart rhythm in a small fraction of AF patients. The combination of high prevalence and limited treatment options imposes significant public health and economic burdens. Therefore, an enhanced understanding of AF pathogenesis and the development of more effective screening methods are imperative [18,19].

TLR4, in conjunction with MD-2, responds to a wide variety of LPS found in the outer membrane of Gram-negative bacteria. These bacteria include members of the *Enterobacteriaceae* family, a heterogeneous group of commonly encountered human pathogens such as *Klebsiella*, *Escherichia*, *Proteus*, *Salmonella*, and *Shigella*. In addition to these, other Gram-negative bacteria such as *Helicobacter pylori*, *Chlamydia trachomatis*, *Neisseria* species, and *Haemophilus* species also seem to play roles in human atrial fibrillation (AF) [5,6,7]. Interestingly, Huang and colleagues have recently found significant differences in the gut microbiota composition between AF patients and controls without AF. In their study, opportunistic pathogenic bacteria like *Klebsiella*, *Haemophilus*, *Streptococcus*, and *Enterococcus* were significantly increased in AF patients, while the levels of certain symbiotic bacteria were significantly decreased [8].

The current data show the key involvement of TLR4 in various aspects of myocardial inflammation across different cardiac conditions [9]. In viral myocarditis (VMC), specifically induced by *Coxsackievirus B3* (CVB3), evidence suggests that TLR4 plays a significant role. Studies in mouse models demonstrate that TLR4-deficient mice exhibit increased resistance to CVB3 infection, showing decreased inflammatory responses, viral replication, and myocarditis compared to wild-type mice [10]. Remarkably, the impact of TLR4 appears to be multifaceted, involving both the MyD88-dependent and MyD88-independent pathways. While TLR4 through the MyD88-dependent pathway contributes to the severity of VMC [9], the MyD88-independent pathway can, in some cases, limit the severity. Also, in myocardial infarction (MI), the release of endogenous damage-associated molecular patterns (DAMPs) from necrotic cardiac myocytes triggers TLR4 activation, leading to the increased expression of pro-inflammatory cytokines, contributing to additional myocardial damage. It is noteworthy that the TLR4 and downstream gene expression profiles are upregulated not only in the infarcted region but also in the remote myocardium post-MI. Inhibition of TLR4 signalling pathways through genetic manipulation or the use of inhibitors like metformin has shown benefits in terms of reducing inflammatory responses and improving cardiac function [11,12]. With regard to myocardial ischemia/reperfusion (I/R) injury, studies performed on mouse models have revealed significant increases in TLR4 and NF-κB expression levels in both the ischaemic zone and the potential danger region [13]. Moreover, TLR4 inhibition by eritoran is able to mitigate the detrimental effects of TLR4 signalling pathways during myocardial I/R injury [14].

In this study, we conducted a meta-analysis to investigate the role of TLR4 in AF using whole-genome expression datasets from the GEO database. Focusing on mRNA expression from the left atrial appendage, we selected five datasets to capture a comprehensive view of gene expression patterns associated with AF. Employing the Fisher inverse chi-square method for meta-analysis, we identified 565 upregulated and 267 downregulated DEGs associated with AF. To explore the TLR4 involvement in AF, we utilized consensus transcriptional profiles from TLR4 knockdown cells obtained from the L1000 database. The analysis highlighted a significant overlap between TLR4 knockdown cells and DEGs associated with AF, providing insights into how TLR4 impacts the transcriptional landscape of this cardiac condition. These results bring to light the complex role of TLR4 in the pathophysiology of AF and the molecular mechanisms involved. To further substantiate these findings and explore the functional implications of TLR4 in AF, additional validation preclinical models that employ genetic manipulation—such as gain-of-function or loss-of-function modifications of TLR4—could significantly advance the understanding of the specific contributions of TLR4 to AF. 

After expanding the analysis to include other TLRs, a substantial number of DEGs exhibited overlapping patterns with the consensus gene signatures of various TLRs. This overlap included both instances of agreement and disagreement in terms of gene regulation. Notably, a more distinct and specific association was identified for TLR2 and TLR3. Specifically, 25 out of 245 downregulated genes upon TLR2 knockdown were shared with DEGs upregulated in AF, and 13 out of 247 downregulated genes upon TLR3 knockdown were shared with AF-upregulated DEGs. The observed association for TLR2 and TLR3 aligns with their expression levels in the cardiac tissue. Specifically, the mRNA expression profile for TLRs in the human heart follows the order of TLR4 > TLR2 > TLR3 > TLR5 > TLR1 > TLR6 > TLR7 > TLR8 > TLR9 > TLR10 [9]. It is noteworthy that the mRNA expression levels for TLR2, TLR3, and TLR4 are approximately tenfold higher compared to TLR1 or TLR5–10 [31]. This differential expression pattern underscores the prominence of TLR2 and TLR3 in cardiac tissues, providing support for the observed correlation in the context of the present study.

On the other hand, the non-specific involvement of MYD88 and TICAM1, as observed in our analysis, aligns with the broader observation that most TLRs other than TLR4 are not specifically associated with AF. Indeed, AF is a complex condition influenced by various factors, including inflammation, oxidative stress, and structural remodelling. Thus, the activation of multiple signalling cascades and pathological events may contribute to the non-specificity observed in relation to MYD88 and TICAM1. Different TLRs are also involved in distinct aspects of the inflammatory response, and the overall inflammatory context in AF could lead to overlapping gene expression patterns. Also, the functional redundancy of TLRs could result in non-specific associations when assessing the impact of individual TLRs on gene expression. Finally, TLR expression patterns can vary among tissues and cell types. In the context of AF, where atrial tissues are of primary interest, the expression patterns of TLRs may not be specifically associated with the differential gene expression observed in AF patients. Consideration of the tissue-specific expression and function of TLRs within the context of AF could provide insights into the specific roles of each TLR.

Our data seem to support a potential use of drugs targeting the TLR4 pathway in the treatment of AF. The effectiveness of TLR4 antagonists and inhibitors has already been demonstrated in animal models of various cardiac diseases. For instance, eritoran, a TLR4 antagonist, proved effective in a murine model of transverse aortic constriction-induced cardiac hypertrophy, showing the reduced production of pro-inflammatory cytokines (such as IL-1β and IL-6) and the increased production of anti-inflammatory cytokines (such as IL-10). A promising TLR4 homolog, RP105, lacking the TIR domain and competitively inhibiting TLR4 signalling, has shown potential in treating cardiac dysfunction after MI. RP105 hampers TLR4 signalling pathways in post-infarction remodelling, conferring protective effects on cardiac function [2]. 

Despite these promising findings, TLR4 inhibitors or antagonists for the prevention or treatment of cardiac diseases have not yet entered human clinical trials. Clinical applications of TLR4 inhibition have been explored in other systems or organs. Notably, the TLR4 antagonist E5564 (eritoran) showed toleration in phase II clinical trials for sepsis treatment. However, it did not result in the desired reduction in 28-day mortality in phase III clinical trials. Possible explanations for this discrepancy include the study design focusing on early-stage sepsis-induced organ dysfunction, the exclusion of patients with established severe sepsis, and differential outcomes in Gram-positive bacterial sepsis. Moreover, TLR4-specific inhibitors like TAK-242 have shown promise in promoting haematoma absorption and improving neurologic deficits following intracerebral haemorrhage. The humanized monoclonal antibody NI-0101 by NovImmune, designed for the treatment of acute and chronic inflammation, is still in the preclinical phase. It selectively binds to an epitope on human TLR4, interfering with the dimerization required for intracellular signalling and induction of pro-inflammatory pathways. Additionally, the viral protein-derived peptide OPN-401, with the ability to inhibit TLR4 signalling, is currently in preclinical development. Furthermore, targeting molecules in the downstream signalling pathways of TLR4 presents a feasible approach [30].

To establish a clearer understanding and ascertain the potential benefits of these drugs, further in vitro and in vivo studies are warranted within the context of AF. These studies should help to elucidate the efficacy of specific drugs and address the multifaceted aspects of AF. It is also noteworthy that the repeated anecdotal clinical observations for drugs with potential to downregulate the TLR4 pathway validate their beneficial effects in AF [32,33,34,35,36], which make the findings of our study of immediate clinical relevance. 

There are several limitations in our study. Firstly, the selection methods of the DEGs, based on the median value and the low fold change threshold, may limit the precision of our results [37,38]. While this approach was chosen to maintain inclusivity during the exploratory phase of our research and to prevent premature exclusion of potentially relevant DEGs, it is acknowledged that it may have increased the likelihood of false positives. Nevertheless, the thresholds used in this study for the selection of the DEGs are commonly used in the scientific literature [39,40,41,42]. Moreover, it is also crucial to note that one of the strengths of our study lies in the use of FDR correction in the selection of DEGs and subsequent functional analysis. By incorporating FDR, we aimed to mitigate the risk of false positives and maintain statistical rigor throughout our analysis. Therefore, while adopting a more stringent FC cutoff might have decreased the number of false positives, it could have also led to an increase in false negatives, potentially overlooking important biological insights.

Secondly, our study lacks a direct comparison of gene expression between the left and right atria, as well as the pulmonary vein, in the context of AF. While the left atrium may have a more pronounced role in the initiation and maintenance of AF [20], our study does not explore these differences. This limitation is noteworthy, as understanding the differential gene expression across these regions could offer deeper insights into the mechanisms of AF and potential therapeutic targets. Future research could be focused on addressing this limitation by specifically examining how gene expression varies between the left and right atria and the pulmonary veins in patients with AF.

## Figures and Tables

**Table 1:** Enrichment of genes associated with atrial fibrillation (AF) within the TLR4 consensus gene signature. Adj *p*-values of < 0.05 are in bold.

	Term	Overlap	*p*-Value	Adj. *p*-Value	Odds Ratio	Combined Score	Genes
Fibrillation Up	TLR4_ko Down	17/243	0.001	**0.001**	2.637	19.7	*PHTF2;JUND;TACSTD2;CXCR4;HSPA2;NXT2;REXO2;ETS1;ZFP36L2;GULP1;PLAC8;RCAN1;MAPKAPK2;ANGPTL2;YPEL5;PDLIM5;S100A8*
	TLR4_ko Up	8/246	0.394	0.395	1.158	1.1	*LYN;NUP50;CCL5;GZMA;ID1;ARHGDIB;DNPH1;SNX5*
Fibrillation Down	TLR4_ko Down	6/243	0.138	0.215	1.755	3.5	*GRB7;TFAP2C;PERP;SOSTDC1;PHLDA2;PPL*
	TLR4_ko Up	9/246	0.009	**0.039**	2.660	12.4	*GRAMD1C;C7;ANXA3;SCNN1A;NR2F1;SLC4A4;SORL1;EREG;DCBLD2*

**Table 2:** Enrichment of genes associated with atrial fibrillation (AF) within the MYD88 and TICAM1 consensus gene signature. Only significant data (Adj. *p* value < 0.05) are shown.

	Term	Overlap	*p*-Value	Adj. *p*-Value	Odds Ratio	Combined Score	Genes
Fibrillation Up	MYD88_ko Down	27/246	1.9 × 10^−9^	2.7 × 10^−8^	4.40	88.4	*CDS1;LST1;CFI;TACSTD2;ITPR1;HBB;TCF21;PLAC8;ALOX5;ARHGDIB;CASP1;HLA-DQA1;LYN;IRX5;CD300A;IGFBP2;RNASE6;LAPTM5;PPBP;MAPK14;NUDT21;TYROBP;SELL;MZB1;B3GNT2;FCGR2C;S100A8*
	MYD88_ko Up	20/243	2.1 × 10^−5^	5.5 × 10^−5^	3.16	34.1	*CFD;COL15A1;VWF;C1S;MTCL1;FHL1;FHL2;DPT;GULP1;AKAP12;ACTA1;MFAP4;EPB41L3;MAPKAPK2;COL5A2;TSPAN4;PDLIM5;CHGB;TGM2;FGFR1*
	TICAM1_ko Down	15/245	4.3 × 10^−3^	5.5 × 10^−3^	2.28	12.4	*UBE3C;FHL2;CYBA;HNMT;MS4A4A;RHOB;MARCO;COL4A2;UST;MZB1;TXNIP;ANGPTL2;CRK;S100A8;CHGB*
	TICAM1 _ko Up	13/244	2.2 × 10^−2^	2.5 × 10^−2^	1.96	7.5	*CD74;PHTF2;ADAM10;CRIP2;SORBS2;HMGB1;GULP1;MFAP4;MECOM;CDH2;TCF4;SKP2;CRYM*
Fibrillation Down	MYD88_ko Down	10/246	3.0 × 10^−3^	2.1 × 10^−2^	2.98	17.3	*CA12;PTPRZ1;FLRT3;CHL1;ANXA3;ANPEP;PLEKHB1;CHI3L1;SORL1;ST6GALNAC5*

**Table 3:** Enrichment of genes associated with atrial fibrillation (AF) within the TLR consensus gene signature. Only significant data (Adj. *p* value < 0.05) are shown.

	Term	Overlap	*p*-Value	Adj. *p*-Value	Odds Ratio	Combined Score	Genes
Fibrillation UP	TLR1_ko Down	24/241	1 × 10^−7^	7 × 10^−7^	3.93	63.4	*BRD2;RAB1A;POSTN;COL15A1;DDX3X;LIMCH1;DUSP1;FHL2;HSPA2;PRSS23;GULP1;NFKBIA;MFAP4;MYO1B;MECOM;OAS1;CCNG2;EPB41L3;ID1;PDK4;PHACTR2;EIF4E2;SRSF10;CHGB*
	TLR1_ko Up	22/245	2 × 10^−6^	8 × 10^−6^	3.49	45.9	*SRGN;ABCC4;RRM1;GYPC;GZMA;LST1;CFI;LAPTM5;TACSTD2;HBB;TCF21;CYBB;CRIP2;PPBP;SORBS1;PRPF4;SELL;RGS1;SUB1;ALOX5;CCL5;S100A8*
	TLR2_ko Down	25/245	3 × 10^−8^	3 × 10^−7^	4.04	69.7	*SPARC;FHL1;HTR2B;HBB;HNMT;FBLN5;MS4A4A;AKAP12;HHEX;PDGFD;ARHGDIB;PDK4;PDE8B;MSR1;ZNF160;SORBS1;PTK2;ACTA1;CXCL12;OAS1;NFIB;MPPED2;NOX4;PLIN2;CRK*
	TLR3_ko Down	13/243	2 × 10^−2^	2 × 10^−2^	1.97	7.6	*CD74;CD300A;CFI;HBB;HSPA2;TMEM135;DHRS9;SELL;OAS1;NAMPT;CASP1;PLIN2;S100A8*
	TLR4_ko Down	17/243	6 × 10^−4^	1 × 10^−3^	2.64	19.7	*PHTF2;JUND;TACSTD2;CXCR4;HSPA2;NXT2;REXO2;ETS1;ZFP36L2;GULP1;PLAC8;RCAN1;MAPKAPK2;ANGPTL2;YPEL5;PDLIM5;S100A8*
	TLR5_ko Down	16/243	2 × 10^−3^	2 × 10^−3^	2.47	15.9	*RERE;COL15A1;SPARC;NLGN4X;TRRAP;IGFBP2;TACSTD2;G0S2;DPT;TFPI;GULP1;AKAP12;SFRP4;PRPF4;ALOX5;PIEZO1*
	TLR5_ko Up	19/247	8 × 10^−5^	2 × 10^−4^	2.93	27.6	*CD52;RAB1A;MEF2C;HPGD;DUSP1;USO1;YIPF5;TNFRSF11B;HK2;RHOB;ACTA1;CARHSP1;MYO1B;LOX;TNNT1;PXDN;ARHGDIB;PHACTR2;SNX5*
	TLR6_ko Down	13/245	2 × 10^−2^	3 × 10^−2^	1.95	7.4	*C1S;RRAD;STAT1;TACSTD2;FHL2;CYBA;GLRX;HSPA2;PTK2;RCAN1;ADAMTS1;NAMPT;GLUL*
	TLR6_ko Up	12/245	5 × 10^−2^	5 × 10^−2^	1.79	5.5	*LAPTM4B;POSTN;MAF;SEMA3C;LMO3;NFATC3;BGN;HMGB1;ETS1;CHGB;PAFAH1B1;TBC1D16*
	TLR7_ko Down	23/243	5 × 10^−7^	2 × 10^−6^	3.71	54.1	*CFD;COL15A1;C1S;PDE1A;FHL1;RNASE6;HNMT;TFPI;CP;MS4A4A;GULP1;PLAC8;MS4A6A;MFAP4;MAF;CXCL12;MDFIC;PDGFD;TXNIP;COL21A1;TCF4;HLA-DQA1;FGFR1*
	TLR7_ko Up	15/242	4 × 10^−3^	5 × 10^−3^	2.31	12.8	*HSPA9;IRX5;BGN;TACSTD2;HK2;RHOB;PRPF4;ALOX5;QPCT;NUP50;ID1;ARHGDIB;GNB1;ITGA8;PIEZO1*
	TLR8_ko Down	20/246	2 × 10^−5^	6 × 10^−5^	3.12	33.1	*CFD;DUSP4;BRD2;DDX3X;NLGN4X;MTCL1;ZNF160;RNASET2;FHL1;TACSTD2;CYBA;ZFP36L2;TBX3;AKAP12;MARCO;SNX1;NFIB;UST;GLUL;TGM2*
	TLR8_ko Up	18/247	2 × 10^−4^	4 × 10^−4^	2.76	22.9	*PSMD10;CD52;CD74;SPON1;POSTN;CISH;LST1;YIPF5;RNASE6;CYBRD1;CRIP2;HNMT;PLAC8;LAPTM4B;MDFIC;LOX;TNNT1;DNASE1L3*
	TLR9_ko Down	30/247	2 × 10^−11^	7 × 10^−10^	4.97	122.6	*CDS1;SPON1;HPGD;SEMA3C;MTCL1;TACSTD2;HNMT;ALOX5;PEA15;TIMM17A;PHACTR2;CRYM;TGM2;YWHAH;CPA3;CD52;BNIP3;HSPA2;SGPP1;RHOB;VEGFA;PTP4A1;MS4A6A;TYROBP;MDFIC;SUB1;QPCT;CCNG2;ID1;CNOT8*
	TLR9_ko Up	22/246	2 × 10^−6^	8 × 10^−6^	3 × 10^0^	45.5	*CFD;BRD2;PDGFRA;MEF2C;COL15A1;ZNF160;PDE1A;FHL1;CYBA;BMP6;HK2;PLAC8;AKAP12;SFRP4;RELN;MAF;CXCL12;PDE8B;ITM2A;KCNJ2;CHGB;FGFR1*
	TLR10_ko Down	25/244	3 × 10^−8^	3 × 10^−7^	4 × 10^0^	70.3	*GLRX;AEBP1;CAMKK2;PHACTR2;SKP2;CRYM;GLUL;LYN;CPA3;BRD2;CD52;AKIRIN1;ANGPT1;DUSP1;ZNF160;IGFBP2;CYBA;CP;SFRP4;RAB14;CCNG2;TXNIP;CTNNB1;TCF4;S100A8*
	TLR10_ko Up	14/246	1 × 10^−2^	1 × 10^−2^	2.1	9.6	*SRGN;CD74;ENTPD1;GYPC;NAB1;TACSTD2;CYBRD1;AKAP12;LAPTM4B;HHEX;CXCL12;SELL;NFIB;RGS1*
Fibrillation Down	TLR1_ko Up	11/245	9 × 10^−4^	1 × 10^−2^	3.32	23.4	*MYBPC1;C7;PTPRZ1;CHL1;ANXA3;ANPEP;GREB1;SCNN1A;CHI3L1;SLC4A4;SORL1*
	TLR4_ko Up	9/246	9 × 10^−3^	4 × 10^−2^	2.66	12.4	*GRAMD1C;C7;ANXA3;SCNN1A;NR2F1;SLC4A4;SORL1;EREG;DCBLD2*
	TLR5_ko Down	12/243	2 × 10^−4^	6 × 10^−3^	3.68	31.1	*SELENBP1;CADPS2;EHF;COL3A1;CHL1;ANPEP;GREB1;NR2F1;NINL;SORL1;SF1;CLGN*
	TLR7_ko Down	9/243	9 × 10^−3^	4 × 10^−2^	2.7	12.8	*AKR1B10;PPP1R1A;CALD1;ANPEP;RUFY3;P2RY14;RUFY2;STC1;AREG*
	TLR7_ko Up	9/242	8 × 10^−3^	4 × 10^−2^	2.71	12.9	*ERBB3;PTPRZ1;ANXA3;CHI3L1;SLC7A11;TUG1;CLDN1;SORL1;MFAP3L*
	TLR8_ko Down	10/246	3 × 10^−3^	2 × 10^−2^	2.98	17.3	*TKTL1;MYBPC1;SEMA6A;FLRT3;ANXA3;ACSL6;KRT7;AQP4;ZNF721;DSC3*
	TLR10_ko Up	10/246	3 × 10^−3^	2 × 10^−2^	2.98	17.3	*TKTL1;BCHE;ARNT2;PTPRZ1;CHL1;ANXA3;KRT7;SLC4A4;EREG;FOSL2*

## Data Availability

All data are available upon reasonable request to the corresponding author.

## Supplementary Materials

The following supporting information can be downloaded at: https://www.mdpi.com/article/10.3390/genes15050634/s1, File S1: Complete list of DEGs.
